# Evolution of the ε and γ phases in biodegradable Fe–Mn alloys produced using laser powder-bed fusion

**DOI:** 10.1038/s41598-021-99042-0

**Published:** 2021-09-30

**Authors:** Črtomir Donik, Jakob Kraner, Aleksandra Kocijan, Irena Paulin, Matjaž Godec

**Affiliations:** Institute of Metals and Technology, Ljubljana, Slovenia

**Keywords:** Materials science, Biomaterials, Biomedical materials, Implants

## Abstract

The key feature of Fe–Mn alloys is gradual degradability and non-magneticity, with laser power bed fusion (LPBF) parameters influencing the microstructure and chemical composition. Our study focuses on biodegradable Fe–Mn alloys produced by mechanically mixing pure metal feedstock powders as part of the LPBF process. The Mn content and, consequently, the γ-ε phase formation in LPBF samples are directly correlated with an adapted energy–density (*E*) equation by combining the five primary LPBF parameters. We varied laser power (*P*) in a range of 200–350 W and scanning speed at 400 and 800 mm/s, and a comprehensive study was performed on samples with similar *E*. The study also showed an almost linear correlation between the LPBF's laser power and the material's hardness and porosity. The corrosion resistance was significantly reduced (from 13 to 400 μm/year) for the LPBF samples compared to a conventionally produced sample due to the dual-phase microstructure, increased porosity and other defects. The static immersion test showed that the process parameters greatly influence the quantity of oxides and the distribution of their diameters in the LPBF samples and, therefore, their corrosion stability. The most challenging part of the study was reducing the amount of ε phase relative to γ phase to increase the non-magnetic properties of the LPBF samples.

## Introduction

Biodegradable metals (BMs)^1–3^ are bioactive materials, and the phase evolution of Fe-based BMs is an area of interest in additively manufactured biomaterials^4–7^. Additive manufacturing (AM), especially the Laser powder-bed fusion (LPBF) technique, has gradually emerged as a powerful platform for personalised medical applications^8–10^. LPBF utilises a high-power-density laser to melt and fuse the metallic powders completely^11–13^. The knowledge gained about the phase evolution of Fe–Mn alloys is essential for a wide range of applications and technologies^14–16^. BMs have a temporary support function with subsequent gradual degradation in vivo by releasing corrosion products when exposed to the physiological environment in the body^17–19^. The research field of biodegradable metallic materials aims to complete the biodegradable implants' dissolution with possible aid to healing the surrounding tissue, with little or no implant residues in the tissue^20–22^. The problem of a too slow in vitro and even slower in vivo degradation rate is often highlighted as the main drawback of Fe–Mn biodegradable implants^23–26^. Due to the wide range of austenite stainless steels (γ) for medical applications^3,27–29^, the problem of phase transformations (α' and ε) in Fe–Mn alloys has never been properly considered and addressed^30–32^. In transformation-induced plasticity (TRIP) steels with Mn^33–35^ as well as in low-density TRIP steels with Mn and Al^36^, the phase transformation from γ-austenite (face-centered cubic, non-magnetic phase) to ε-martensite (hexagonal close-packed, paramagnetic PM phase) and α'-martensite (body-centered cubic, ferrimagnetic FM phase) were studied in depth. These kinds of steels show a deformation-induced phase transformation from γ-austenite to ε-martensite and α'-martensite or directly from γ-austenite to α'-martensite. Phase transformations in Fe–Mn BMs are crucial due to the different magnetic properties of the phases and were studied for the different effects, i.e., the shape-memory effect, where the reverse transformation of stress-induced ε-martensite to γ-prior austenite occurs upon heating. Lee et al.^35^ studied the influence of the volume fraction of each constituent phase with Mn content in the as-quenched state for Fe–Mn alloys. At 28 wt.% of Mn, the only constituent phase was γ-austenite.

Although there has been extensive research on BMs, no single study exists with a focus on the magnetism aspect of the BM's Fe-phases and their influence on medical applications and broader areas^37^. When magnetic metallic biomaterials (MMBs) are exposed to the extremely high magnetic field in a magnetic resonance imaging device, unfavourable heat effects can occur. MMBs in the body interact with the strong static, gradient magnetic field and the radio-frequency (RF) electromagnetic pulses used during MR scanning. This interaction of the magnetic fields used in MR scanners causes disturbances such as magnetically induced force, torque, and tissue heating due to the RF's coupling with the MMBs, which can seriously injure people with such implants^37–39^. Even the latest review paper dealing with Fe–Mn BMs failed to adequately address ^40^ the issue of magnetic problems in BMs.

The final properties of the LPBF-produced parts are greatly influenced by scan-related and temperature-correlated process parameters^12,13,41^. Analyses of those parameters and their influences on the mechanical, microstructure, or corrosion properties are most often related to the density of the material. Pekok et al.^42^ conducted experiments that varied *P*, scanning speed (*v*), and hatch distance (*H*), where the relative and Archimedes' densities were considered. These densities are directly related to the porosity (a higher porosity contributes to a lower density). A higher porosity, which results from an increased reaction surface, leads to faster corrosion and can be achieved with process parameters that deviate from the optimum (too high or too low *E*)^43^. In the study of Liverani et al.^44^, the increased corrosion rate of CoCr LPBF-produced samples was achieved with a lower *E* value. The same relationship between porosity and corrosion rate was reported by Qian et al.^45^ for a Ti6Al4V alloy with the same *P* value*.* The relative density decreased with an increasing *v*. As expected, faster corrosion was observed for the highest value of *v* (lowest density = highest porosity).

This study focuses on developing biodegradable Fe–Mn alloys from the mechanical mixing of elemental feedstock powders via the LPBF process, with the aim to achieve gradual degradability, non-toxicity and non-magnetic properties. Our challenge was to select the appropriate LPBF parameters to obtain the desired chemical composition and, consequently, phases in the microstructure. It is essential that any material implanted in the human body allows all medical diagnostics and therefore needs to consist of non-magnetic phases or utmost traces of paramagnetic material. Therefore, understanding the relationship between the LPBF parameters and the microstructural development is crucial to achieving the appropriate corrosion and mechanical properties for successful implementation in biomedical applications.

## Materials and methods

### Material

The feedstock of pure Fe and Mn powders (Goodfellow) was mixed to form two powders with 67.0 wt.% and 33.0 wt.% of Fe and Mn, respectively. A Shaker Mixer TURBULA® Type T2 C (Willy A. Bachofen AG) was used to prepare the powder mixture. The mixing lasted for 1 h before the experiments. Fe powder was irregularly shaped with a size of 45 ± 15 μm and Mn powder was angularly shaped with a size of 30 ± 15 μm. The flowability of mechanically mixed powders was consequently very low, proven with a 49 ± 2° angle of repose. For the chemical analyses, an Agilent 720, with the ICP-OES technique, was used to determine the compositions of the Fe–Mn alloys (Table 1).

**Table 1:** Sample labelling with process parameters and final chemical compositions (wt.%).

Sample	Laser power *P* (W)	Scanning speed *v* (mm/s)	Energy density *E* (J/cm^3^)	Fe (wt.%)	Mn (wt.%)
S-350/400	350	400	1215	79.3 ± 0.4	20.5 ± 0.2
S-300/400	300	400	1042	78.1 ± 0.5	21.5 ± 0.2
S-250/400	250	400	868	77.3 ± 0.4	22.3 ± 0.3
S-200/400	200	400	694	76.0 ± 0.4	23.5 ± 0.2
S-350/800	350	800	608	75.8 ± 0.5	23.8 ± 0.3
S-300/800	300	800	521	75.5 ± 0.4	23.9 ± 0.2
S-250/800	250	800	434	74.8 ± 0.4	24.8 ± 0.3
S-200/800	200	800	347	74.7 ± 0.4	24.7 ± 0.2

### Process parameters and models

The AconityMINI LPBF equipment allows control of the laser power (*P*), scanning speed (*v*), and laser diameter (*d).* The *P* values varied between 200 and 350 W, and the *v* values were set to 400 mm/s and 800 mm/s. The laser diameter (*d*), hatch distance (*H*) and layer thickness (*t*) values were constant: *d* = 100 μm, *H* = 80 μm, *t* = 30 µm and with scanning strategy of 54°. The cube-shaped models with dimensions of 10 × 10 × 10 mm^3^ were produced to prepare process maps. The Vickers micro-hardness was measured with an Instron Tukon 2100B with 1 kgf. For the potentiodynamic measurements, 5-mm-high and 20-mm-diameter cylinders were produced, while smaller cubes (0.5 mm × 0.5 mm × 0.5 mm) were manufactured for the gravimetric corrosion measurements.

### Microscopy and mechanical measurements

For the light microscopy (LM), a ZEISS Axio Imager Z2m was used, and scanning electron microscope (SEM) analyses were performed on a CrossBeam 550 ZEISS FIB-SEM with a Hikari Super electron backscatter diffraction (EBSD) Camera from EDAX. For the EBSD measurements, a 70° tilt angle with an acceleration voltage of 15 kV and a probe current up to 10 nA was used. A larger area (approximately 2 × 2 mm^2^) was examined on each selected sample, and at least three EBSD mappings (170 × 220 μm^2^ each) were measured. The most representative parts were chosen for this investigation.

Electron channelling contrast imaging (ECCI) was used to reveal the cellular dislocation structure. With this technique, where the electrons are backscattered on the crystal defects, and under certain conditions, the defects are shown as white features on a dark background. The ECCI was performed at 30 kV and a probe current of 2 nA.

The samples for SEM analyses using the EBSD and ECCI techniques were mechanically polished with 1-µm diamond suspension, followed by 10 min of OPS (SiO_2_ nanoparticles) finishing.

### Corrosion measurements

The corrosion measurements of the additively manufactured samples were performed using electrochemical potentiodynamic measurements. Studied samples with an exposed 1 cm^2^ were prepared for the electrochemical testing in standard Hank's solution (8 g/L NaCl, 0.40 g/L KCl, 0.35 g/L NaHCO_3_, 0.25 g/L NaH_2_PO_4_(2H_2_O, 0.06 g/L Na_2_HPO_4_(2H_2_O, 0.19 g/L CaCl_2_(2H_2_O, 0.41 g/L MgCl_2_(6H_2_O, 0.06 g/L MgSO_4_(7H_2_O, 1 g/L glucose, pH = 7.8). All the samples were mechanically prepared with SiC paper up to 4000 grit and polished to a mirror finish. Before each measurement, the samples were washed with acetone and deionised water, finished by air drying. All the solutions for the experiment were prepared using MERK chemicals with deionised water. For the electrochemical measurements, a three-electrode system was used. A Fe–Mn electrode was used as the working electrode, a Pt mesh as the counter electrode, and a saturated calomel electrode (SCE) as the reference electrode. A Potentiostat/Galvanostat BioLogic SP 300 (France) with EC-Lab V11.27 software was used for all the potentiodynamic polarisations with a scan rate of 1 mV/s. The data were gathered to compare the electrochemical parameters, i.e., corrosion current density *i*_corr_, corrosion potential *E*_corr_ and corrosion rate *v*_corr_, to compare the differently prepared samples.

Static immersion tests were performed in 0.1 M lactic acid at 37 °C for 7 days and in Hank's solution at 37 °C for 30 days. The concentration of dissolved species was measured after the immersion tests using inductively coupled plasma optical emission spectroscopy (ICP-OES, Agilent 720).

## Results and discussion

**Figure 1:**
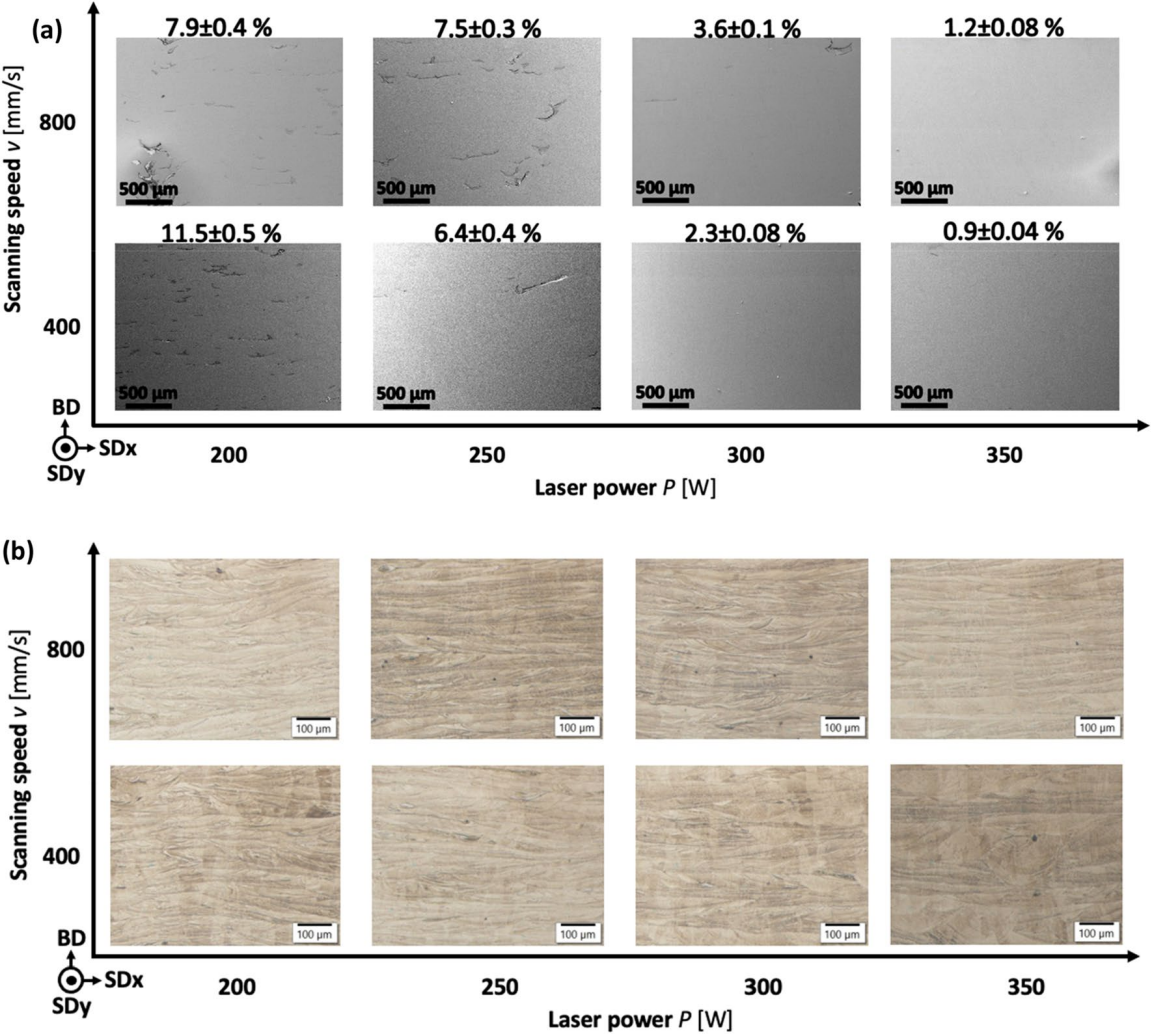
SE (**a**) and LM (**b**) images of the samples with the amount of defects (porosity, oxides and impurities).

Systematically arranged Secondary Electron (SE) and LM images of samples produced with various *P* and *v* values are presented in Fig. 1 as two process maps. Observed defects are more common in the samples from the left side of the process map (Fig. 1a). These defects are a consequence of too low *P* values for constant and stable metal-powder melting. According to scanning strategy of 54° the laser path is almost never perpendicular to sample cross-section. Therefore, melt-pool shape presentation is changing depending on laser path. LM etched microstructures revealed that the melt-pool shape changed according to *P* value (Fig. 1b). At scanning speed 400 mm/s the size of melt-pools increases by higher laser power. Larger melt pools indicate larger thermal impact zone. At higher scanning speed (800 mm/s) this phenomena is not so distinct, with flaten melt-pools’ shape.

The process parameters *P*, *d*, *v*, *H* and *t* are the variables that determine the final properties of the material. These process parameters are all combined in the equation for energy density (*E*). Regardless of the selected additive-manufacturing technique, the *E* value is the most relevant parameter. The most cited equations for calculating *E* use energy per unit area or volume^46^. Prashanth et al.^47^ reported an equation that considers four essential parameters. The reliance on laser power *P*, scanning speed *v*, layer thickness *t*, and hatch distance *H* results in a value for *E*, but the laser's diameter *d* is not included. The *d* can also vary with *H*, and the Eq. ^47^ is appropriate only for the same *d* and *H* values, so there is a need for an equation that simultaneously considers values for *d* and *H*. An equation that ignores the *H*-to-*d* ratio relative to the other three process parameters only gives the correct calculation for the energy density (*E*) in the case of equal *H* and *d* values. With the introduction of the ratio between *d* and *H*, which are always directly associated, in the Eq. ^11^, the *E* calculation covers all five crucial operational parameters at the same time using the equation:

$$E = \frac{P \cdot d}{{v \cdot H^{2} \cdot t}}$$

**Figure 2:**
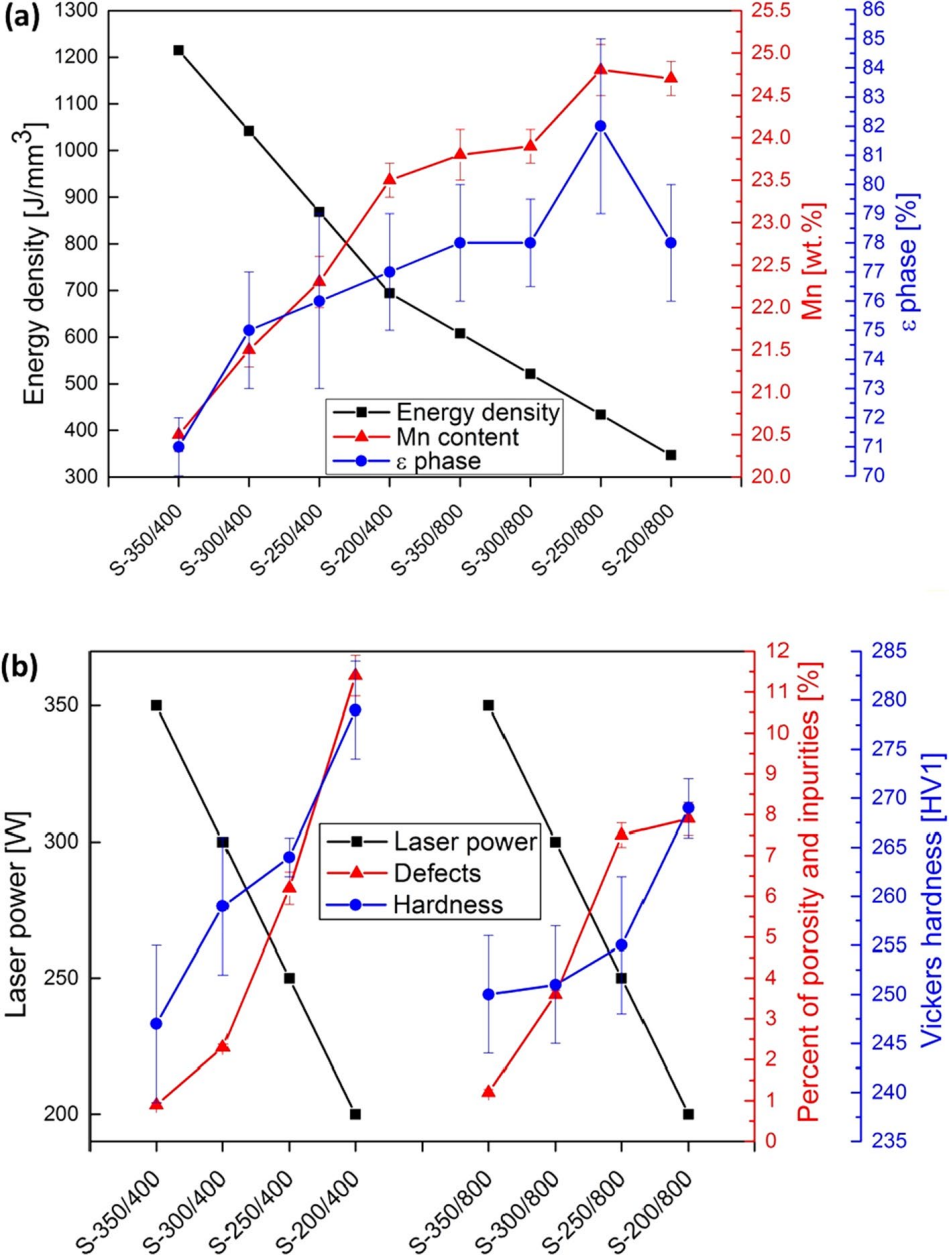
Correlation of the concentration of Mn and ε phase with the LPBF parameter *E* (**a**) and correlation of the percentage of impurities and material hardness with the LPBF parameter *P* (**b**).

The graphical presentation in Fig. 2 supports the process-map description and explanation. Four different *P* and two different *v* values were compared as varied process parameters with different *E*. With a decrease of the *E* values, the amount of Mn in the alloys increases (Fig. 2a). The lower Mn losses due to vaporisation correlate directly with the lower *E* values, contributing to the targeted chemical composition. With the Mn content increased up to 25 wt.% in S-250/800, the ε phase is proportionally increasing and achieves its highest amount, i.e., 80%. This composition can be considered the highest capacity for the ε-phase transformation for our LPBF-produced samples. The ε-phase transformation is directly correlated to the amount of Mn, which can be observed in Fig. 2a.

Figure 2b presents the correlation of the LPBF-produced samples with the associated *P* values. With increasing *P* values, the number of defects (porosity, oxides and impurities) and the studied samples' hardnesses decrease accordingly. The results also show that higher *P* values cause a stronger tempering effect, which occurs during the LPBF process due to the thermal impacts caused by the melting of each next layer and consequently lowers hardness. Thermal effects, such as quenching, tempering and annealing, partially influence the crystal-grain-growth mechanism. However, the grain size, which correlates with the hardness, is challenging to evaluate in a two-phase γ-ε microstructure. It is known that the higher amount of martensite increases the material hardness; however, this impact is not predominant in our study. This trend is much more linear for a lower *v* value (400 mm/s) than for a higher value (800 mm/s). These results confirmed that the number of defects in the material, its hardness, the amount of Mn, and the ε phase are correlated and dependent on more than just one process parameter.

**Figure 3:**
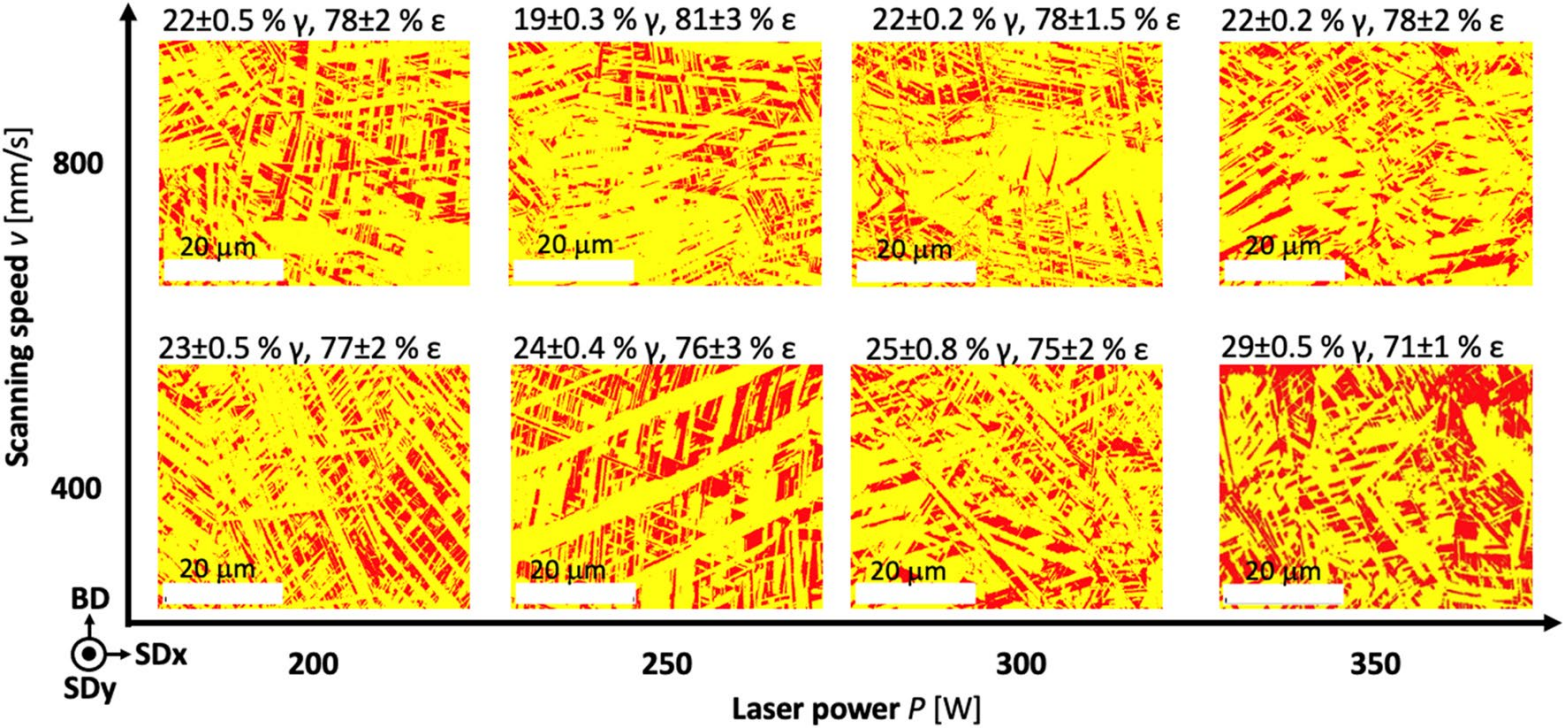
Phase maps of ε phase—yellow, and γ phase—red for LPBF samples produced at different process parameters.

Figure 3 presents the EBSD phase analyses process map, which reveals the highest amount (81 ± 3%) of ε phase in the sample S-250/800 with 20.5 wt.% of Mn content and the lowest (71 ± 1%) amount of ε phase in the sample S-350/400 with 24.8 wt.% of Mn, obtained in our study. Previous studies^35,48,49^ have shown that the amount of Mn significantly impacts the ε-γ phase transformation. Lee et al.^35^ somehow disregarded the C content's importance since their amount of C was around 0.02 wt.%, and they achieved only γ phase already at 28 wt.% Mn. Mesquita et al.^48^, on the other hand, considered and included the carbon content, which influences the phase transformation for Fe–Mn alloys. Our previous studies^49–51^ correlate with Mesquita et al.^48^, where we obtained the only γ phase already at 17 wt.% of Mn with 0.58 wt.% C. Therefore, the amount of ε and γ phases in our current study, with carbon-free material, also fits with the results of Mesquita et al.^48^. However, we could not achieve only γ phase due to production problems (glass and chamber contamination and consequently laser-beam scattering) related to the Mn evaporation with an increased amount of Mn in the feedstock. Our study increased the Mn content in the feedstock up to 53 wt.%, but we obtained only 32 wt.% of Mn in the LPBF sample, the microstructure of which still consisted of ε and γ phases.

**Figure 4:**
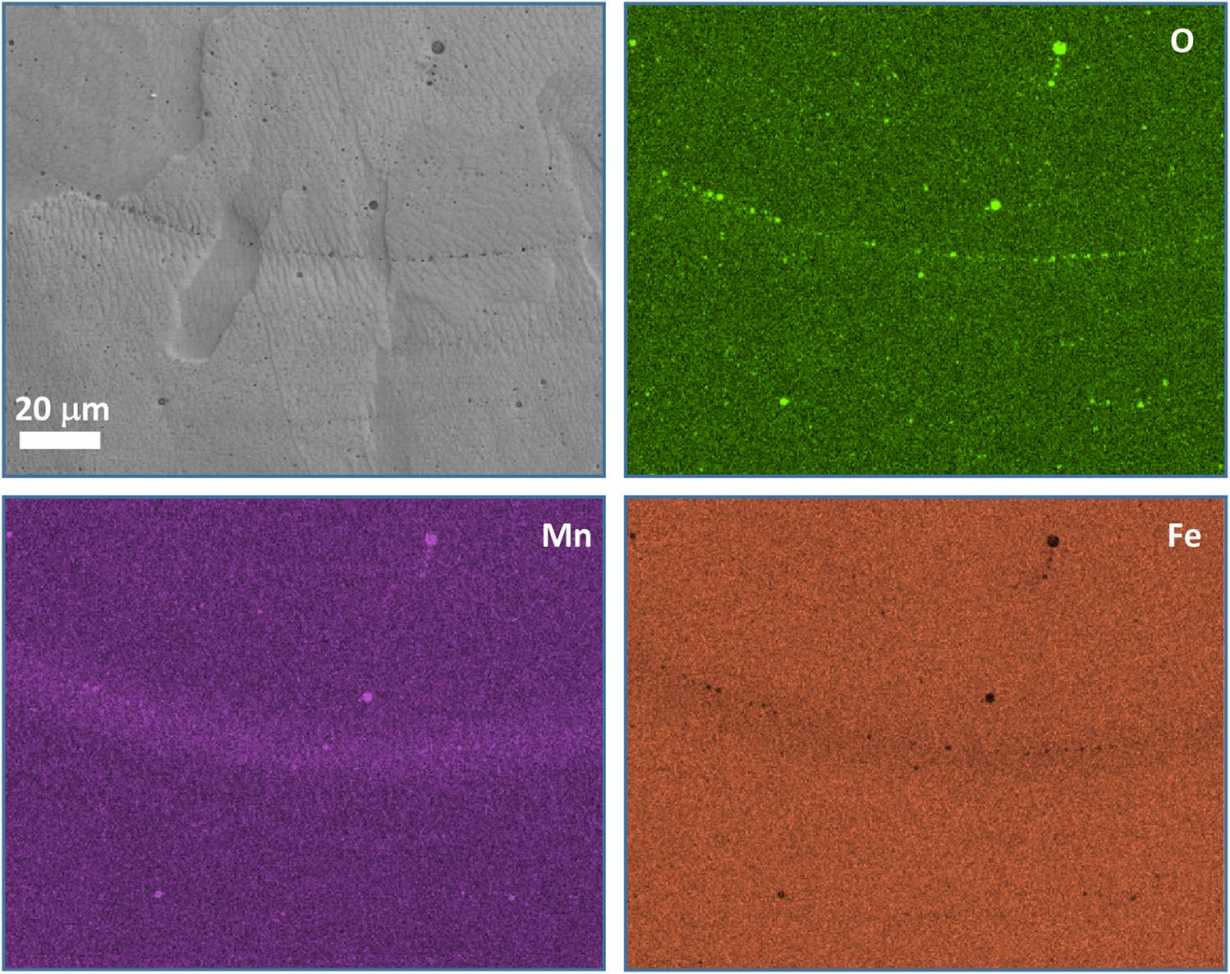
SEM–EDS mapping of Mn, O and Fe at the bottom of the melt pool with clearly visible small Mn oxides on the melt-pool boundary in the S-350/800 sample.

The SEM–EDS mapping presented in Fig. 4 was used to detect segregations and different microstructural phases in the S-350/800 sample. Mn oxides were observed along the melt-pool boundaries. The concentration of Mn is detected in the area surrounding the melt-pool edge. The solidification of Mn oxides (Mn_x_O_y_), due to the sequence in our ternary system, is completed before the solidification temperature for γ_Fe,Mn_ occurs^52^. Thermodynamically, the formation of Mn oxides has a Δ*G* that is more than 200 kJ/mol lower than the Fe oxides, as presented in the Ellingham-Richardson diagram. Consequently, no Fe oxides were found in the analysed microstructure. However, fast cooling from the melt-pool edges into the centre of the melt pool^53^ leads to the highest amount and the arrangement of Mn oxides on the melt-pool boundary^54^. The highest amount of Mn at the melt-pool boundary can be ascribed to the same reason^55^. The interval of high-temperature exposure during the LPBF process is the shortest at the melt-pool boundary and the surrounding area^55^. On the other hand, the areas closer to the melt-pool centre are exposed to more extended periods of high temperatures, which can cause higher vaporisation of Mn. Additional Mn losses can be expected from the higher probability of re-melting the melt pool's upper part than the bottom part closer to the melt-pool boundary^56^.

**Figure 5:**
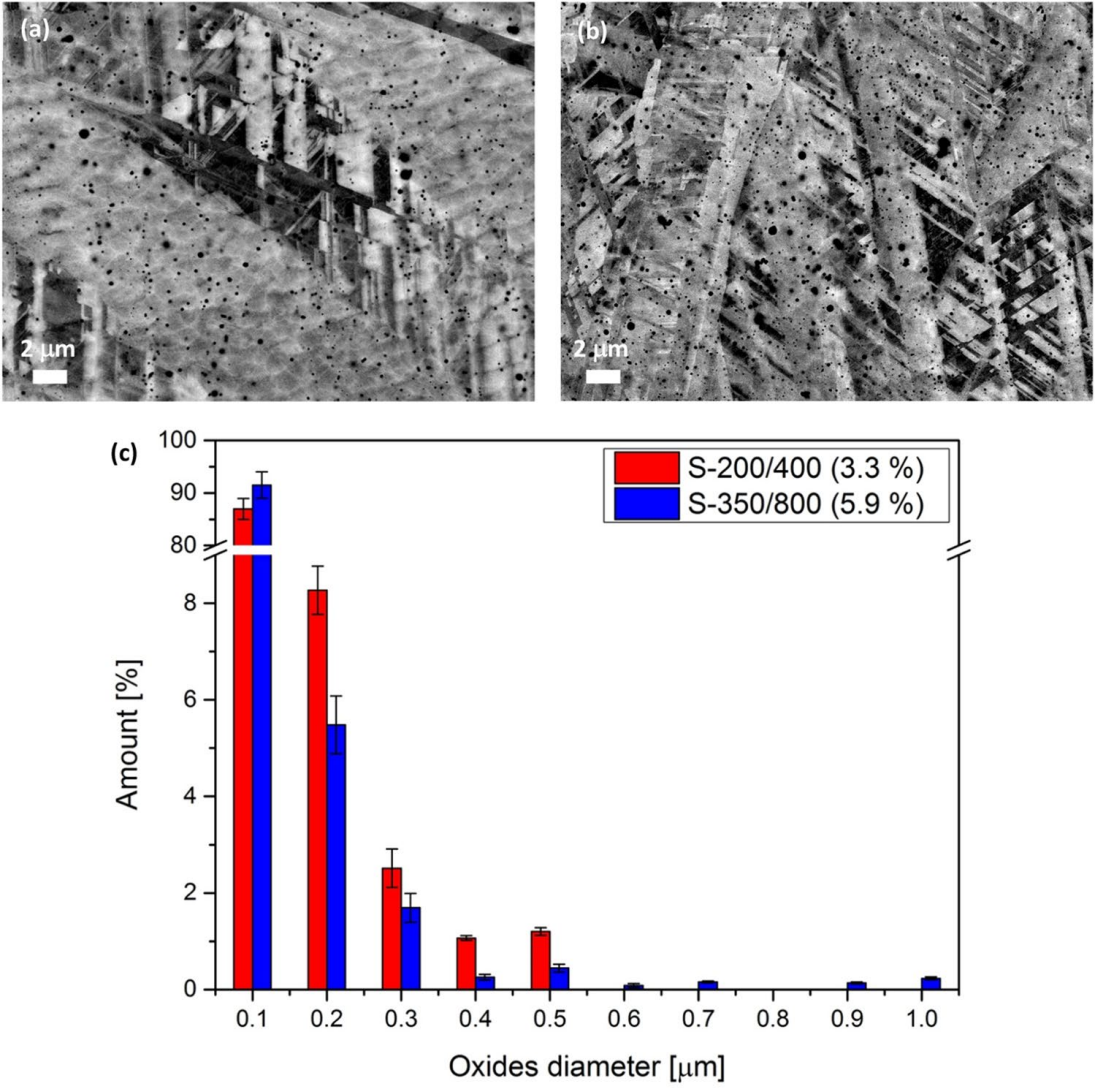
ECCI images of samples S-200/400 (**a**) and S-350/800 (**b**) with different amounts and sizes of small oxides in the matrix with its histogram of the diameters of the oxides (**c**).

LPBF samples with various processing parameters show the oxides' size and distribution in the ECCI micrographs for S-200/400 and S-350/800, respectively (Fig. 5a and b). Generally, the difference between the samples is in the amount and distribution of oxides. The volume ratio of oxides in sample S-200/400 is 3.3%, compared to 5.9% in sample S-350/800, representing almost double the amount of oxides. Besides the significant increase in oxides' formation, the higher *P* value also resulted in a different distribution of the oxides' diameters. As presented in Fig. 5c, the distribution of the oxides' diameters for S-200/400 is from 0.1 μm, where the highest percentage is achieved for both samples, to 0.5 μm. A significantly larger range of oxides' diameters is observed for the sample S-350/800. The smaller number of oxides with diameters of 0.9 μm and 1.0 μm were observed in the sample produced with the highest *P* value. The observed oxides' origin can be ascribed to the elemental Mn powder, which contained some traces of oxide and from the native oxide on the Mn and Fe powders. This presence of oxides in AM stainless steel^57,58^ is well known and has a beneficial impact on the mechanical properties of the produced parts. Process parameters such as the *P* and *v* impact the melt's temperature and melt-pool dynamics, which combined influence the size and distribution of oxides.

**Figure 6:**
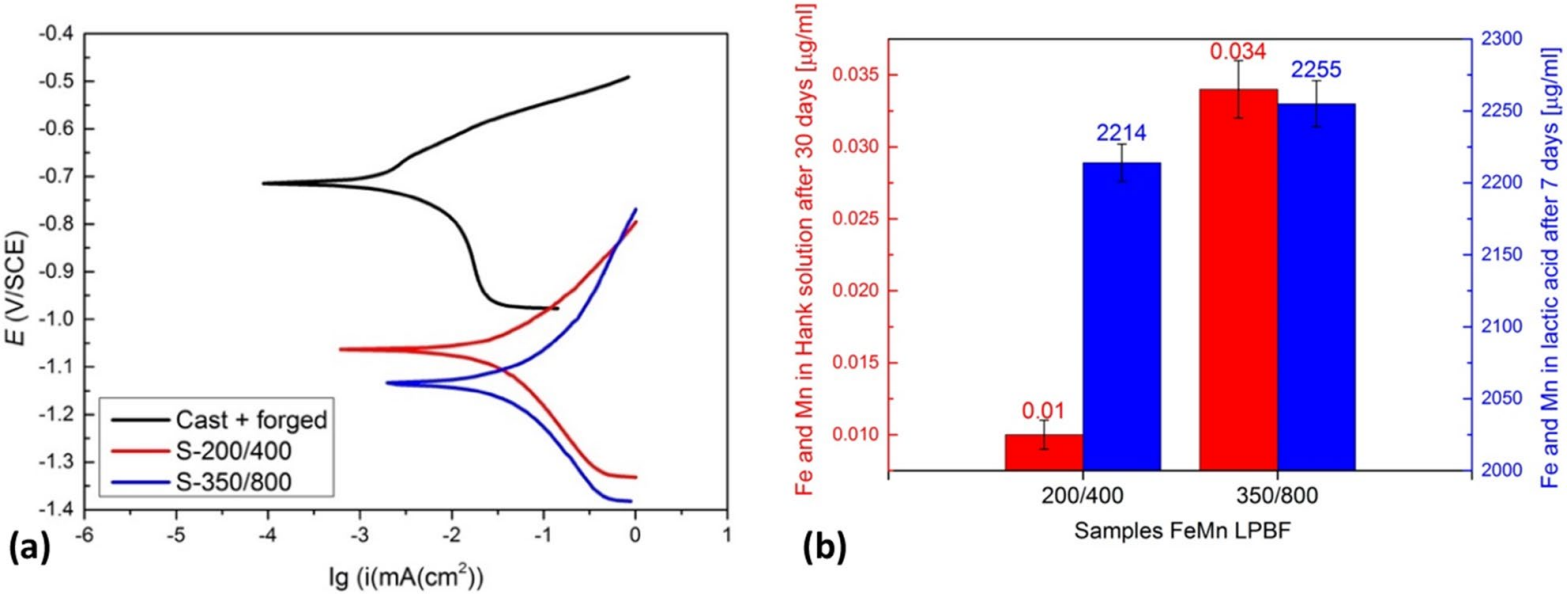
Corrosion results with potentiodynamic measurements of samples S-200/400 and S-350/800 compared with the cast and forged sample with a similar chemical composition measured in Hank's solution (**a**) and total concentration of Fe and Mn after the static immersion tests of samples S-200/400 and S-350/800 performed in 0.1 M lactic acid and Hank's solution at 37 °C (**b**).

Potentiodynamic polarization curves of samples S-200/400 and S-350/800 compared to a cast-and-forged sample with a similar chemical composition measured in Hank's solution are presented in Fig. 6a. The calculated values of the corrosion rates (*v*_corr_), corrosion current densities (*i*_corr_) and corrosion potentials (*E*_corr_) are listed in Table 2. A significant increase of *v*_corr_ and *i*_corr_ was observed for both LPBF samples compared to the cast-and-forged sample. Therefore, the decreased corrosion resistance of the LPBF samples can be mainly ascribed to the increased porosity compared to the conventionally produced sample. Furthermore, reduced corrosion stability in LPBF material can be correlated to dual-phase microstructure (γ-austenite and ε-martensite) in comparison to conventionally produced material which consists of austenitic phase only. In LPBF material, more crystal lattice defects, dislocations, grain boundaries, retain stresses and oxides were observed, significantly reducing corrosion resistance^16^.

**Table 2:** Corrosion parameters calculated from Tafel region.

Sample	*E*_corr_ (mV)	*i*_corr_ (μA/cm^2^)	*v*_corr_ (μm/year)
Cast + forged	− 710 ± 5	1.1 ± 0.1	13 ± 1
S-200/400	− 1074 ± 8	34.3 ± 0.7	408 ± 9
S-350/800	− 1138 ± 9	34.4 ± 0.7	409 ± 9

On the other hand, the potentiodynamic measurements did not show any significant differences between the S-200/400 and S-350/800 samples, regardless of whether they differ substantially in terms of the amount of oxides and their diameter distribution. As already mentioned, the volume ratio of oxides in the sample S-350/800 was twice that in the sample S-200/400 and their diameter distribution (Fig. 5). Nevertheless, these results contributed to the corrosion behaviour of both LPBF-manufactured samples under static immersion conditions. Furthermore, the S-350/800 sample's increased dissolution was observed compared to the S-200/400 sample regarding the influence of the different process parameters (Fig. 6b).

## Conclusions

This study focuses on developing biodegradable Fe–Mn alloys from the mechanical mixing of elemental feedstock powders via the LPBF process, analysing the properties of gradual degradability, non-toxicity and non-magneticity. Any material implanted in the human body must enable all medical diagnostics. Therefore, it needs to consist of non-magnetic phases or only trace amounts of paramagnetic material.

The LPBF produced samples' chemical compositions were correlated with an adapted energy–density equation combining all five LPBF parameters. This equation allows us to tailor the Mn content, known for its low evaporation pressure, from an initial 33.0 wt.% to a final 20.5% up to 24.8 wt.% in the LPBF-produced sample. The study also showed an almost linear correlation between the LPBF laser power and the material hardness and porosity. In addition, the decreased corrosion resistance was proven for all the investigated LPBF materials due to the increased oxide content and the higher porosity compared to conventionally produced material with the same chemical composition.

This study has contributed to our understanding of ε-phase formation in Fe–Mn BMs and the limitations related to the production of carbon-free alloys in LPBF processes. The most challenging part is the elimination of the ε phase to achieve the non-magnetic properties of the LPBF-produced samples. The main drawback lies in the severe chamber contamination with excessive Mn feedstock and the inability to add carbon, which both enable γ stabilisation. In our ongoing research we therefore propose replacing the mechanically mixed elemental feedstock powders with Fe–Mn–C alloyed powders to avoid extensive Mn-evaporation problems during production. Further work is also needed to fully understand the implications of a combination of alloy and elemental feedstock powders.
